# TECHNICAL VARIATION IN AFFIXING HAMSTRING GRAFTS TO THE TIBIA IN ACL RECONSTRUCTION

**DOI:** 10.1590/1413-785220182602155160

**Published:** 2018

**Authors:** MARCUS VINICIUS DANIELI, JOÃO PAULO FERNANDES GUERREIRO, ALEXANDRE OLIVEIRA QUEIROZ, CARLOS ROBERTO PADOVANI

**Affiliations:** 1. Hospital Uniorte and Hospital Santa Casa de Londrina, Londrina, PR, Brazil.; 2. Instituto de Biociências da Unesp de Botucatu, Botucatu, SP, Brazil.

**Keywords:** Anterior cruciate ligament reconstruction, Tendons, Orthopedic fixation devices., Reconstrução do ligamento cruzado anterior, Tendões, Dispositivos de fixação ortopédica

## Abstract

**Purpose:**

To present a technical variation in tibial fixation of quadruple hamstring grafts during anatomic reconstruction of the anterior cruciate ligament (ACL). The secondary purpose was to decrease the costs associated with this procedure.

**Methods:**

Twenty patients who underwent ACL reconstruction were selected. A tibial tunnel was constructed using standard techniques, and a femoral tunnel was anatomically created using the outside-in technique. The hamstring autograft was passed (with its bend) into the tibial tunnel and affixed to the tibia using the suspensory technique and a simple staple. Femoral fixation was performed using a titanium interference screw. The patients underwent postoperative evaluations at 0, 3, 6 and 12 months using the subjective International Knee Documentation Committee (IKDC) form and Lysholm knee scores.

**Results:**

The IKDC and Lysholm score results improved over time (p<0.001) without major complications. The cost of the procedure could be reduced by using lower-cost hardware (staples).

**Conclusion:**

The proposed technique for anatomic ACL reconstruction using inverted hamstring grafts with their bend in the tibial tunnel, suspension-type fixation using a staple demonstrated good to excellent results after 1 year of follow up, with lower aggregate costs. Level of Evidence IV; Case series.

## RESUMO

**Objetivo:**

Apresentar uma variação técnica na fixação do enxerto quádruplo de tendões flexores à tíbia na reconstrução anatômica do ligamento cruzado anterior (LCA). O objetivo secundário é diminuir o custo associado ao procedimento.

**Métodos:**

Foram selecionados 20 pacientes que seriam submetidos à reconstrução do LCA. O túnel tibial foi realizado usando técnica padrão, e o túnel femoral foi realizado pela técnica anatômica de fora para dentro. O enxerto (tendões flexores autólogos) foi passado com sua dobra dentro do túnel tibial e fixado com um agrafe simples pela técnica de suspensão. A fixação femoral foi realizada com parafuso de interferência de titânio. Os pacientes foram avaliados no pré-operatório e aos 3, 6 e 12 meses pós-operatórios usando o questionário International Knee Documentation Committee (IKDC) subjetivo e o questionário de Lysholm.

**Resultados:**

Os resultados dos escores dos questionários IKDC e Lysholm tiveram sempre melhora com o tempo (p < 0,001) sem maiores complicações. O custo total da cirurgia foi reduzido usando material de custo menor (agrafe).

**Conclusão:**

A técnica proposta para reconstrução anatômica do LCA com enxerto de tendões flexores invertido com sua dobra no túnel tibial, com fixação tipo suspensão com um agrafe, mostrou bons a excelentes resultados após 1 ano de acompanhamento, com menor custo agregado. Nível de Evidência IV; Série de casos.

## INTRODUCTION

Anterior cruciate ligament (ACL) injuries are frequent in active young people and can potentially cause instability and reduce knee function.^1^ Surgical treatment is recommended when patients complain of instability and to prevent associated injuries.^2^ This treatment is so widely accepted that approximately 100,000 ACL reconstructions are performed each year in the United States,^3^ and more than 90% of these surgeries yield good to excellent results.^4^

Cournapeau et al.^5^ showed that much of the costs of ACL reconstruction are related to disposable arthroscopy materials and implants; the high incidence of this procedure consequently raises concerns about its costs. In Brazil, the Unified Health System (Sistema Único de Saúde, SUS) pays R$ 486.00 for a titanium interference screw (source: personal contact with SUS suppliers, checked against payment receipts on November 29, 2017). Using the data on incidence in the United States as an example (since these data are not available in Brazil), the total cost of using two interference screws in each ACL reconstruction is approximately R$ 97.2 million per year.

Until recently, the most common technique used in ACL reconstruction was based on tunnel isometry,^6^ creating the femoral tunnel through the tibial tunnel. However, this technique does not place the graft in the original anatomical position of the ACL.^6^ ^-^ ^9^ Placing the ACL graft in the original position can restore the original anatomy and biomechanics of the ligament, and this technique has been shown more effective in stabilizing rotational movements of the knee.^6^ ^,^ ^8^

This study proposes anatomic reconstruction of the ACL using flexor tendons, creating an outside-in femoral tunnel. In the tibia, a variation of the graft fixation technique uses the principle of suspension with a simple staple, reducing the cost of surgery. The objective of this study is to demonstrate that this technique is simple, reproducible, effective, and involves lower aggregate costs.

## MATERIALS AND METHODS

The study was approved by the institutional review board (record No. 381/11), and all patients signed an informed consent form before inclusion in the study.

Between January 2011 and January 2012, 20 patients agreed to participate in the study. Inclusion criteria were patients between 18 and 45 years old with ACL injuries treated at the authors’ outpatient clinic. Patients were excluded if they had arthritis, previous surgery, deformity, or associated injuries to other ligaments in the affected knee, or any injury to the other knee.

**Figure 1:**
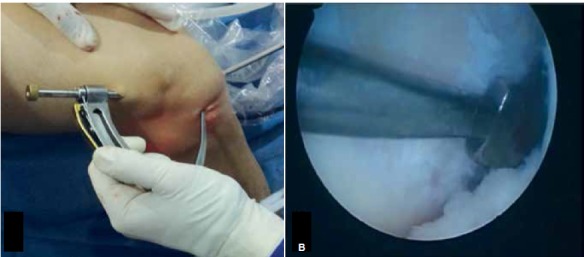
Tibial guide for the ACL, placed through the anterolateral portal to create the outside-in anatomic femoral tunnel (A). Intra-articular view (B). Right knee.

**Figure 2:**
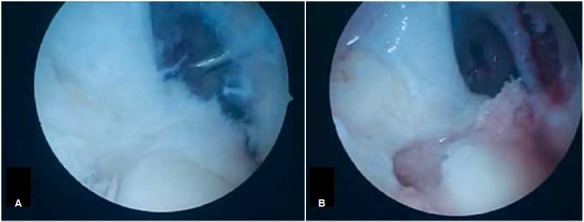
Femoral guide wire placed using the outside-in technique (A). Femoral and tibial tunnels - arthroscopic view (B). Left knee.

**Figure 3:**
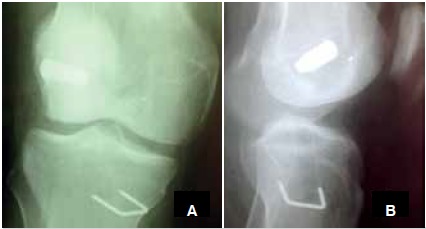
Postoperative X-rays: (A) AP and (B) lateral views showing tibial fixation with staple (right knee).

**Figure 4:**
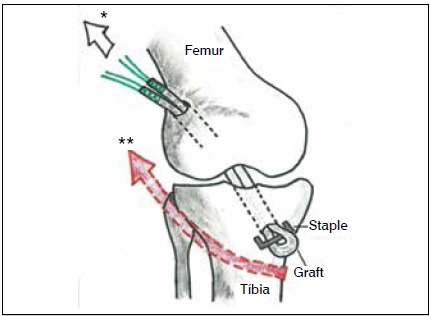
Illustration showing tibial fixation of the graft with a staple (inverted graft and suspension technique) in a right knee. Graft traction (*) and reduction of anterior draw (**).

Surgical Technique: After spinal anesthesia, the semitendinosus and gracilis tendons were removed using the standard technique. Arthroscopy is then performed, and associated injuries are treated if necessary. Next, with the knee at 90º flexion, the standard tibial guide for ACL reconstruction was positioned in the center of the remaining tibial ligament through a medial portal at a 55º angle, and a guide wire inserted. A tunnel measuring 8 to 9 mm (according to the thickness of the graft) was then drilled. After this, with the knee remaining at 90º flexion, the camera was inserted through the medial portal and the same standard ACL tibial guide was inserted through the lateral portal to create the femoral tunnel in an outward-to-inward direction. (Figures 1 and 2) The guide was held at a 60º angle; the entry is approximately 2 cm proximal and 2 cm anterior to the lateral epicondyle of the femur, as described by Lubowitz et al.^10^ Next, a guide wire was introduced and a tunnel created with the same diameter as the one in the tibia. The graft was passed from the femur to the tibia, with the fold facing downward, and subsequently attached to the tibia using the suspension technique at the tunnel exit and a simple, smooth staple. Note that the staple does not compress the graft against the bone, but only fixates the suspension. (Figure 3) Traction was then exerted on the graft with the knee at 30° flexion (to “pull” the tibia and reduce the anterior draw), (Figure 4) and the graft was then attached to the femur with an interference screw from the outside in.

Standard rehabilitation protocol was used in all patients, with immediate therapy recommended. Partial load with crutches was permitted for 10 to 15 days after surgery, and patients were evaluated at 10 days. Pain, knee-related symptoms, physiotherapy protocol, range of motion, stability (anterior drawer test, pivot shift, Lachman, valgus and varus), meniscal symptoms, limb alignment, and overall function were evaluated monthly.

Subjective IKDC (International Knee Documentation Committee) and Lysholm questionnaires were applied during the pre-operative period and at 3, 6, and 12 months post-surgery, and scores were calculated and recorded.

Multivariate analysis of variance was used for the repeated measurement model, along with Bonferroni’s multiple comparison test,^11^ considering 5% significance.

## RESULTS

Mean patient age was 29.95 years, and the sample was composed of 17 men and 3 women. A total of 12 right knees and 8 left knees were included. Associated injuries were as follows: 14 cases of medial meniscal injury, 7 lateral meniscus injuries, 7 patients with chondral injury, and 3 with isolated ACL injury. (Table 1) 

**Table 1:** Age, sex, side, and associated injuries for each patient.

Patient	Age	Sex	Side	Associated injuries
1	18	M	R	MM
2	20	M	R	MM + LM + Chondral
3	19	M	R	MM + Chondral
4	35	M	L	MM + LM + Chondral
5	40	M	R	MM
6	21	M	L	Chondral
7	42	M	L	MM + Chondral
8	30	F	R	LM
9	31	M	R	
10	35	M	L	MM + LM + Chondral
11	45	M	L	MM
12	23	F	L	
13	34	M	R	MM + LM
14	17	M	L	MM + Chondral
15	34	M	R	LM
16	25	F	R	MM
17	36	M	R	
18	43	M	L	MM + LM
19	21	M	R	MM
20	30	M	R	MM

Three patients (15%) were lost to follow-up (two prior to 3 months post-procedure and 1 after 3 months). The data for these patients were excluded.

The results (scores) for the IKDC and Lysholm questionnaires at 0, 3, 6, and 12 months are shown in Tables 2 and 3. The results showed significant improvement over time (p<0.001).

**Table 2:** IKDC questionnaire scores (mean and standard deviation) according to evaluation time.

		Evaluation Time			
	Pre-op	3 m post-op	6 m post-op	12 m post-op	P
IKDC	41.65 (4.74)	58.65 (3.11)	86.94 (2.08)	90.88 (2.30)	p<0.001

**Table 3:** Lysholm questionnaire scores (mean and standard deviation) according to evaluation time.

		Evaluation Time			
	Pre-op	3 m post-op	6 m post-op	12 m post-op	P
Lysholm	63.00 (5.75)	80.26 (7.87)	90.89 (5.14)	94.61 (2.79)	p<0.001

In terms of complications, two patients had limited extension (3º and 5º) compared with the normal side. However, this limitation did not compromise results during the evaluation period. 

All patients returned to their pre-injury activity levels without major complaints such as pain, instability, insecurity, or muscle deficit. No patient had graft rupture as of the time of the last evaluation.

## DISCUSSION

During the first 4 weeks after ACL reconstruction, graft fixation is the weak link, and bone density plays an important role in this factor.^12^ Because its bone is spongy and denser than the tibia, fixation of the femur generally presents greater resistance.^13^ Additionally, femoral fixation may be transverse or suspension-type, both of which are more resistant than the techniques more commonly used in the tibia, which in turn mostly involve compression with interference screws.^14^ ^,^ ^15^ This weakness can be compensated by fixation using suspension in the tibia, thus increasing resistance of the fixation in the immediate postoperative period, which is essential for safe rehabilitation and to allow the graft to integrate. This study demonstrated that this is possible using simple fixation material.

The technique described was possible because of the inverted folds in the graft, placing it within the tibial tunnel. This option was first described by Howell and Taylor,^16^ but even though these authors also used simple fixation materials, they described a more laborious type of graft fixation.

During anatomic reconstruction of the ACL, the femoral tunnel can be created by either the medial or medial accessory portals (transportal technique) or from the outside in.^7^ ^,^ ^9^ ^,^ ^17^ ^-^ ^20^ Cadaver studies have shown both techniques to be biomechanically similar.^7^ ^,^ ^8^ ^,^ ^18^ ^,^ ^19^ For the transportal technique, the tunnel must be created with the knee at approximately 110º flexion, and the medial femoral condyle should be protected to prevent a short tunnel and chondral injury.^17^ The outside-in technique has the advantage of better accuracy in positioning, with less risk of rupturing the posterior cortex of the femur (blow-out).^7^ ^,^ ^8^ ^,^ ^17^ ^,^ ^20^ The disadvantage of this technique is cosmetic, the need for an additional yet small incision.^7^ ^,^ ^17^ The outside-in technique was selected in this study, and none of the patients complained of the extra scar.

Another important technical detail is that the graft is first fixed to the tibia. It is then pulled, and finally secured to the femur. The advantage is reduction of anterior draw without the need for other maneuvers, because the graft transmits the traction and pulls the tibia. (Figure 4) This fixation sequence is theoretically more logical and biomechanically superior. However, this superiority must be confirmed through future biomechanical studies.

Cost of the procedure is an important factor due to the high incidence of this type of surgery. The most common fixation method in the Brazilian public health system (SUS) utilizes titanium interference screws (one for the tibia and another for the femur). SUS pays R$ 486.00 for each of these screws, and R$ 25.00 for a simple, smooth staple; as a result, replacing one of these screws with a staple saves R$ 461.00 per surgery. This practice reduces the final cost of the procedure and contributes to the country’s economy.

### Limitations

First, even though one year is a short follow-up period, the main objective of this study was to prove that the technique is easy and effective, with good to excellent results. Although the sample size was small, the results showed significant improvement over time. Another weak point is the absence of a control group. Sample size was also not calculated. Objective results were not presented, but all patients who completed the follow-up returned to their pre-injury activities without pain, instability, or graft rupture, and none required additional surgery.

## CONCLUSIONS

The proposed technique for anatomic ACL reconstruction, using an inverted autologous quadruple flexor tendon graft with the fold within the tibial tunnel fixed with the suspension technique and a staple, and an anatomic femoral tunnel created with the outside-to-inside technique, showed good to excellent results in this series of 20 cases, with lower material costs. Additionally, this technique can be reproduced with common materials available for ACL surgery.
